# Effects of various pre-slaughter weights on the physico-chemical qualities of pig meat

**DOI:** 10.5455/javar.2021.h542

**Published:** 2021-09-25

**Authors:** Mykola Povod, Olekasndr Mykhalko, Oleksandr Kyselov, Victor Opara, Valery Andreychuk, Yevheniia Samokhina

**Affiliations:** 1 Department of Feed Technology and Animal Feeding, Sumy National Agrarian University, Sumy, Ukraine; 2 Department of Biochemistry and Biotechnology, Sumy National Agrarian University, Sumy, Ukraine; 3 Department of Technology of Livestock Production of Polesie National University, Zhytomyr, Ukraine; 4 Department of Breeding and Selection of Animals and Water Bioresources, Sumy National Agrarian University, Sumy, Ukraine

**Keywords:** Color intensity, marbling, meat quality, pre-slaughter weight, water holding capacity

## Abstract

**Objective:**

The article aimed to study the relationship between the physico-chemical qualities of pig meat and their pre-slaughter weights.

**Materials and Methods:**

In this study, 60 pigs were reared for fattening under the same conditions of keeping and feeding, slaughtered, and used to sample the longest back muscle meat with pre-slaughter weights of 110 and 130 kg. The samples were evaluated according to generally accepted methods for assessing the physico-chemical qualities in pig meat.

**Results:**

Samples of meat from animals slaughtered at 130 kg had higher values for marbling – by 2.0 points or 5.24% (*p* < 0.01), active acidity pH in ham muscles – by 0.20 pH or 3.57% (*p* < 0.01), and the longest muscle of the back – by 0.10 pH or 1.82% (*p* < 0.001). The pre-slaughter weight factor substantially affected the pH of ham muscles at 10.35% and on the marbling of meat in the longest back muscle at 13.31%. Pigs slaughtered at 110 kg had a greater increase in the color intensity of the meat and an increase in its water holding capacity. At a pre-slaughter weight of 130 kg, increasing the marbling and decreasing the softness of the flesh demonstrated a modest, adverse relationship.

**Conclusion:**

The findings support the use of pre-slaughter weight management to enhance pork quality.

## Introduction

In pig production, slaughter weight is a management variable that affects production costs and product quality. Slaughter weight is a significant economic element since it affects the productivity and profitability of pig processing and the pork quality. Over the last few decades, marketing weight has increased globally, owing to the dilution of fixed production costs through increased weights per pig and the enhancement of genetic selection for lean-type pigs. Wu et al. [1] described an increase in industrialization that enabled scale economies [2]. Meat and meat products provide the body with full proteins, lipids, vitamins, minerals, and extractives necessary for biological synthesis and energy costs. Simultaneously, both meat processors and consumers place a premium on meat quality.

According to Bankovska [3], the meat quality of Ukrainian pigs is determined by the genotype factor at a higher degree (21.5%–73.0%) than the live weight factor (2.1%–19.6%). Combining innovative technology for growing and fattening pigs with a one-sided selection bias during the development of new breeds and kinds has a substantial impact on the quality and production of meat and the growth and ratio of plastic material in animals. Intensive selection modifies the pig’s metabolic and technological aspects of meat: structure, color, water holding capacity, chemical composition, fat distribution pattern, flavor and aromatic attributes, and loss during heat treatment [4]. Franco [5] showed that both pre-slaughter weight and genotype had a substantial effect on the stiffness characteristics of pig flesh, but an examination of its texture profile revealed considerable changes between samples, primarily due to genotypic differences.

According to Li et al. [6], the carcass traits and meat quality are determined by the pigs’ pre-slaughter weight, not their genotype. The primary aspect that confers a competitive advantage on each pork producer is the quality of the pig carcasses, which is mainly determined by their weight [7].

At the same time, it is worth noting that the consumer attractiveness of pig corpses, as determined by initial visual assessment by consumers, is dependent on pre-slaughter live weight and increases considerably (*p* < 0.05) as live weight climbs to higher weight categories (125 kg) [8].

According to Cobanovic et al. [9] and Czyak-Runowska et al. [10], pigs weighing between 115 and 130 kg had less meat and a higher prevalence of pale, soft, and exudative and dark, hard, and dry meat when compared to equivalents with a lower pre-slaughter weight. According to Elbert [11], increasing slaughter weight is associated with increased sensory scores for flavor, juiciness, and acceptability (*p* < 0.05). Similarly, Rice et al. [12] showed that pork from carcasses weighing more than 125 kg received higher desirable (*p* < 0.05) evaluations for appearance and intention to purchase than swine weighing 111 kg. Rice et al. [13] also noted that the juiciness of pork with a slaughter weight of 124 kg was evaluated to be greater (*p* < 0.05) than meat from both 119 and 111 kg pig carcasses.

However, recent research [14–17] reveals that increasing pre-slaughter weight did not affect the pH, color, moisture retention ability, or tenderness of pork and other indices. Milczarek et al. [18] confirmed the lack of a statistically significant effect of pig pre-slaughter weight on the physico-chemical characteristics of meat, with the exception of intramuscular fat content, which was highest in bigger experimental animals. According to Van den Broeke et al. [19], carcass weight had no effect on the meat quality in pigs, but carcass quality improved with a larger pre-slaughter weight in surgically castrated and immunocastrated boars. As the global pig industry continues to expand, it is critical to understand how pre-slaughter weight influences the carcass value of today’s pigs selected for lean growth and those picked for meat quality.

For the preceding decade, there was substantial research on the effect of pre-slaughter weight on the quality of pig carcasses, economic indicators of fattening, and the growth rate of pigs. Furthermore, scientists’ findings of the effect of pre-slaughter weight on the physico-chemical properties of meat were inconsistent and even contradictory. Recent years have seen a decline in such investigations, despite the rapid advancements in pig breeding technologies. Thus, the understudied effect of pigs’ pre-slaughter weight on meat quality is one of the pressing challenges of further research into ways to boost the pig industry’s efficiency and find more genetic potential sources for Irish meat in Ukrainian pig farms.

Recently, the effect of pre-slaughter weight on meat quality has resurfaced as major genetic corporations conduct extensive selection to improve the meat content of pig carcasses. Increased meat content in pig carcasses enables pigs to be fattened for a longer period of time without increasing their fat content and, thus, without affecting feed conversion. The purpose of this study is to determine the effect of pigs’ pre-slaughter weight on the dynamic behavior of physico-chemical parameters and the chemical composition of pig flesh. We hypothesized that increasing pre-slaughter weight would have a beneficial effect on the physico-chemical quality of pig meat.

## Materials and Methods

### Biological material

On the basis of Globinsky Pig Complex Limited Liability Company (LLC)’s fattening farm No-3, Poltava region, Ukraine, we conducted an experiment to determine the relationship between the meat quality of pigs and their pre-slaughter weight. We took pigs from local F1 sows of Irish Ladras and Yorkshire that had been artificially inseminated with boar sperm from the Max Gro synthetic terminal line. A group of 400 pigs was formed from the selected animals with an equal number of boars and pigs and fattened at the age of 70 days.

### Feeding and rearing conditions

During the fattening of the herd, 60 pigs were maintained in identical conditions in 40 m^2^ group pens with a slotted concrete floor and entire meals fed in liquid form.

### Sampling

Individual animals were weighed when they achieved an average weight of 120–125 kg. According to the results of this weighing, 30 heads weighing 110–130 kg were chosen for control pre-slaughter. Before killing, the animals were placed on a starvation diet with unlimited access to water for 24 h. All procedures followed Council Directive 86/609/EEC [20] on the protection of animals used in research and other scientific endeavors. After 24-h fasting, the control slaughter was conducted using the generally acknowledged method [21] at the Globinsky Meat Factory LLC in the Poltava region of Ukraine. After slaughter, the carcasses were chilled to a temperature of 2°C–4°C for 24 h. While deboning the carcasses, samples of the longest back muscle at the level of 9–12 thoracic vertebrae were taken from pigs of both weight categories to determine their physico-chemical properties and chemical composition. These analyses were conducted in the certified laboratory of the Globinsky Meat-packing Plant LCC, Poltava region, Ukraine.

### Analysis of the physical indicators

The color intensity of the meat was judged visually by a six-member trained panel using a 1–5 point score (1 = no, 5 = abundant) on the Minolta L* (Canada) color scale, which had an image that accurately reflected the surface color of the longest back muscle.

Muscle marbling was visually assessed for the presence, density, and size of connective tissue veins in a section by a six-member trained panel using a 1–5 point score (1 = no, 5 = abundant) of the color scale Minolta L* (Canada), which included an image of the exact structure of the veins on the surface of the back’s longest muscle.

The water holding capacity was determined using a modified version of Grau and Hamm’s technique [22]. In general, 500 mg meat samples from each treatment were crushed for 3 min in a filter-press system. The water holding capacity of duplicate samples was determined [Eq. (1)] as a ratio of the sample film area to the overall area as follows:

1 $$\mathrm{WHC}=\frac{\mathrm{Meat}\;\mathrm{area}({\mathrm{mm}}^{2})}{\mathrm{Total}\;\mathrm{area}({\mathrm{mm}}^{2})}\times 100$$

Tenderness of the meat was also determined by pressing according to Grau and Hamm [22] by recalculation [Eq. (2)] according to Severiano [23] as follows:

2 $$X=\frac{S\times 100}{0.3}$$

where *X* is the tenderness of meat cm^2^/gm of total nitrogen; *S* is the area of pressed meat (obtained by determining the water holding capacity); and 0.3 is the total nitrogen content in a portion of meat, %.

After slaughter, meat pH values were determined after 15 min (pH 1.5), 24 h (pH 2.4), and 48 h (pH 4.8) using a pH meter Testo 205 (AG, Germany). Prior to measurement, the pH meter was calibrated in line with ISO 2917:1999 [24] on buffers with known pH.

The electrical conductivity (EC_24_) of meat was measured 24 h after slaughter using a PH/PT-STAR conductometer (R. Matthaus, Germany).

### Analysis of the chemical indicators

The bound moisture content was determined using method Severiano [23] [Eq. (3)] as follows:

3 $$B=\frac{(A-8,\times A)\times 100}{M}\times 100$$

where *B* is the content of bound water (% to meat); *A* is the total water content in the sample (mg);

*B* is the wet spot area (cm^2^); and *M* is the portion of meat (mg).

Determination of water content in meat was carried out in accordance with ISO 1442:1997 [25] and calculated [Eq. (4)] from the difference between the weights of the samples before and after drying as follows:

4 $$X=\frac{(m1-m2)\times 100}{(m1-m)}\;\times 100$$

where *X* is the moisture content, %; *m*^1^ is the weight of the sample with the byux before drying, gm; *m*^2^ is the mass weights with a byux after drying, gm; and *m* is the byux mass.

The following devices were used to determine the moisture content of meat: electric drying box 2B-151 (from 40°C to 200°C, at 50°C ± 5°C; at 200°C ± 2°C); drying box Memmert UF 110 (Germany, with ambient temperature up to 300°C); electronic laboratory scales AS 220C (1 class ± 1 mg from 0.01 to 220 gm); electronic laboratory scales AS 220\X (1 class 1 mg from 0.01 to 220 gm).

The protein content was evaluated using the K-437 digest system and the Kjeldahl sample method in accordance with ISO 5983-1:2005 [26]. The following devices were used to conduct the evaluation: laboratory electronic scales AS 220\X (1 class 1 mg from 0.01 to 220 gm); heating units with programmable temperature profiles DKL 8°C ± 0.5°C ( ±0.2% of full scale) from 10°C to 450°C, Velp Scientifica, Italy; and a device for steam distillation (distillation) UDK 129 (Velp Scientifica, Italy).

The fat content was determined using the Soxhlet extraction method as specified in ISO 6492:1999 [27] with the following instruments: electronic laboratory scales AS 220C (1 class 1 mg from 0.01 to 220 gm); drying cabinet Memmert UF 110 (Germany, with ambient temperature to 300°C); solvent extractor complete with SER 158/6 (Velp Scientifica, Italy); sample hydrolyzer prior to fat determination, six positions (Velp Scientifica, Italy).

### Statistical analysis

The received data were statistically analyzed (Excel 2010) using well-established statistical procedures and one-factor variance analysis. They were expressed as mean values, standard deviations, and standard error of the mean in tables. Student’s *t*-test was used to determine the significance of differences (*p* ≤ 0.05, *p* ≤ 0.01) between the means of chemical composition and physico-chemical attributes in pork (*n* = 30). Additionally, the correlation coefficients of a linear mathematical model were obtained using the least squares method.

### Ethical approval

We took adequate measures during the trial to reduce pain or discomfort in the experimental pigs. Experiments were conducted in compliance with the International Committee on Animal Ethics’ recommendations. The experiment methodology was approved by the Sumy National Agrarian University Bioethics Commission for Animal Care and Use in Scientific (Experimental) Research (ethical approval number BT-21-0215-01). Animals were cared for and used strictly in compliance with Ukraine’s Law No 3447-IV, 2006, on protecting animals from harsh treatment, and Ukraine’s Law No 692, 2008, on the humane treatment of animals.

## Results

The research findings (Table 1) indicate that pigs with a pre-slaughter weight of 110 kg are likely to outperform those of 130 kg in terms of physical and chemical carcass characteristics: water holding capacity of meat and softness. Additionally, there was a tendency to diminish the color intensity of the meat, the free and bound moisture content, and the fat and moisture content in 110 kg pigs.

Simultaneously, significantly higher values were observed in carcasses of animals weighing 130 kg prior to slaughter compared to 110 kg peers in the control group: marbling – by 2.0% or 5.24% (*p* < 0.01), active acidity pH 2.4 on 24 h after slaughter in ham muscles – by 0.20 pH or 3.57% (*p* < 0.01), and in the longest back muscle – by 0.10 pH or 1.82% (*p* < 0.001).

There was no significant difference between pigs of both groups in other physico-chemical qualities of meat.

**Figure 1.**
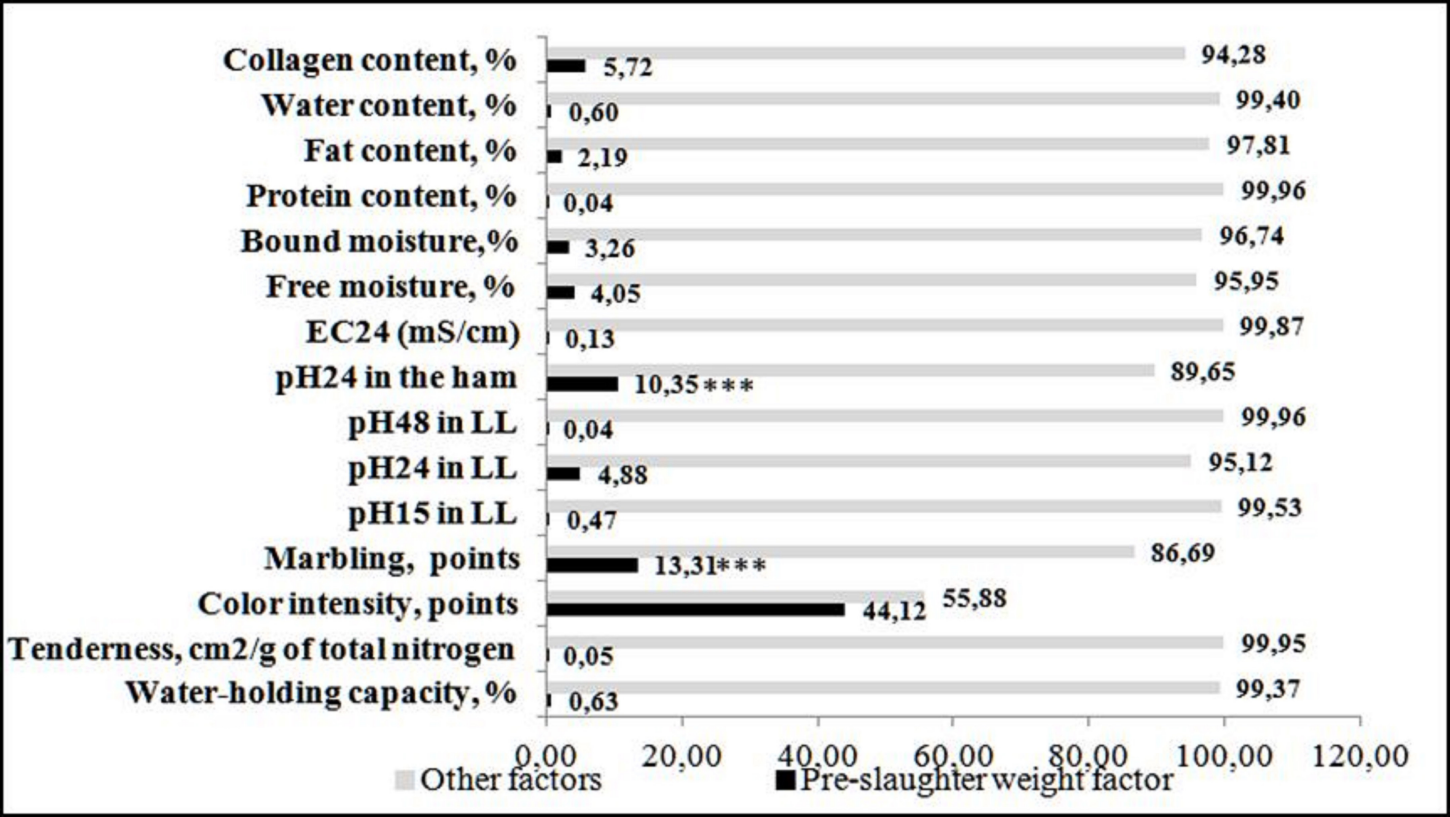
The strength of the influence of the pre-slaughter weight factor on the physico-chemical parameters of carcasses. ***p <0.001.

The findings of a one-factor analysis of variance revealed that the majority of indicators of pig carcass physico-chemical characteristics did not have a direct statistically significant relationship with the pre-slaughter weight (Fig. 1).

It was established that the pre-slaughter weight factor had a significant effect on the active acidity pH in ham muscles 24 h after the slaughter at 10.35% (*F*-value 6.46 > *F*-critical 4.01) and on the marbling of meat in sections of the longest back muscle of pigs at 13.31% (*F*-value 8.60 > *F*-critical 4.01). Simultaneously, unexplained causes caused these indices to fluctuate by 89.65% and 86.69%, respectively.

We employed the least squares approach to examine the presence and strength of a linear association between meat color and the degree of active acidity pH in the longest back muscle 24 h after slaughter in animals weighing 130 kg pre-slaughter and analogs weighing 110 kg pre-slaughter.

The established pairwise correlation coefficients (*r*_*x,y110*_ = −0.4604 тa *r*_*x,y*__130_ = −0.3278) indicated a moderate (0.3 < *r*_*xy*_ < 0.5), inverse (*r*_*x,y*_ < 0), and statistically significant association (*F*-value > *F*-critical) between these indicators for animals in both weight groups, i.e., as the pH of the meat lowers, the severity of its reddening increases slightly in both groups.

**Table 1.** Physico-chemical quality indicators of pig carcasses.

Indicators	Pre-slaughter weight		
	Group I (110 kg)	Group II (130 kg)	Significance
Water holding capacity, %	5.0 ± 0.18	4.8 ± 0.14	NS
Tenderness, cm^2^/gm of total nitrogen	1,047.5 ± 16.12	1,043.3 ± 18.68	NS
Color intensity, points Minolta L*	13.7 ± 0.40	14.5 ± 0.42	NS
Marbling, points Minolta L*	38.2 ± 0.44	40.2 ± 0.52	**
pH_15_ in LL	6.2 ± 0.04	6.2 ± 0.06	NS
pH_24_ in LL	5.5 ± 0.01	5.6 ± 0.01	***
pH_48_ in LL	5.5 ± 0.02	5.5 ± 0.03	NS
pH_24_ in the ham	5.6 ± 0.05	5.8 ± 0.07	**
EC_24_ (mS/cm)	12.4 ± 0.17	12.4 ± 0.20	NS
Free moisture, %	12.8 ± 1.56	16.2 ± 1.60	NS
Bound moisture,%	39.5 ± 1.48	41.6 ± 0.49	NS
Water content, %	72.2 ± 1.52	73.2 ± 1.06	NS
Protein content, %	23.4 ± 0.08	23.4 ± 0.07	NS
Fat content, %	2.0 ± 0.11	2.1 ± 0.11	NS
Collagen content, %	0.7 ± 0.02	0.8 ± 0.02	***

NS – Not significant, LL – Musculus longissimus lumborum.

* *p* <0.05,

** *p* <0.01,

*** *p* <0.001.

**Figure 2.**
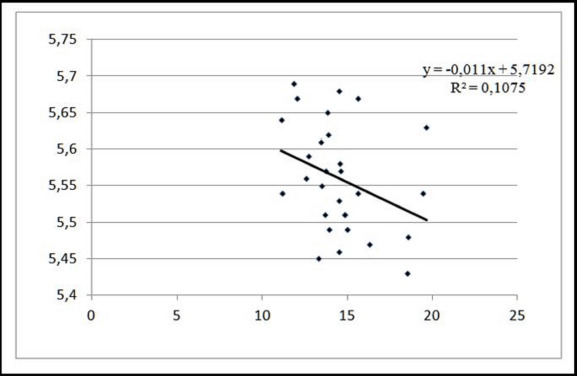
Linear approximation of the dependence of meat color intensity on the pH level at the pre-slaughter live weight of 130 kg.

The coefficient of determination reveals that for pigs weighing 130 kg before slaughter, changes in the effective trait (color intensity) were shaped by the factor trait’s behavior (its pH level) up to 10.75%, while the remaining changes were controlled by random factors (Fig. 2). According to the inverse linear regression equation coefficient, for each 1.0 point of color darkening in the musculus longissimus lumborum (LL) at a pre-slaughter weight of 130 kg, the level of active acidity pH increases proportionately by 0.011 points on the Minolta L* color intensity scale.

**Figure 3.**
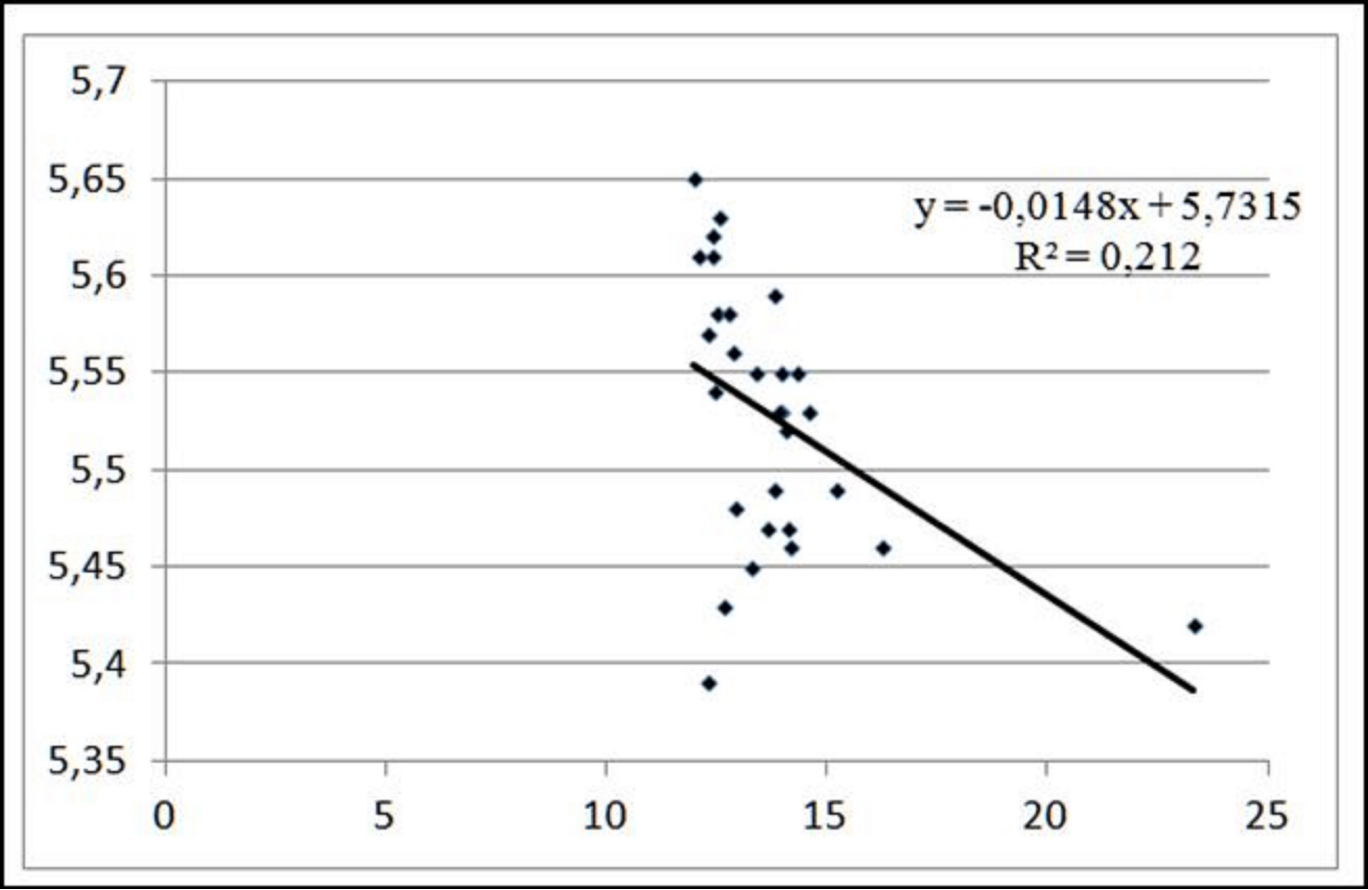
Linear approximation of meat color intensity dependence on the pH level at the pre-slaughter live weight of 110 kg.

The coefficient of determination indicates that the change in meat color intensity was caused by a change in its pH level of 21.20% for pigs with a pre-slaughter weight of 110 kg and that other variations were produced by unaccounted causes (Fig. 3). Similarly, an increase in the amount of redness of 0.014 points was found when the level of active acidity was decreased by 1.0 pH in group I pigs.

**Figure 4.**
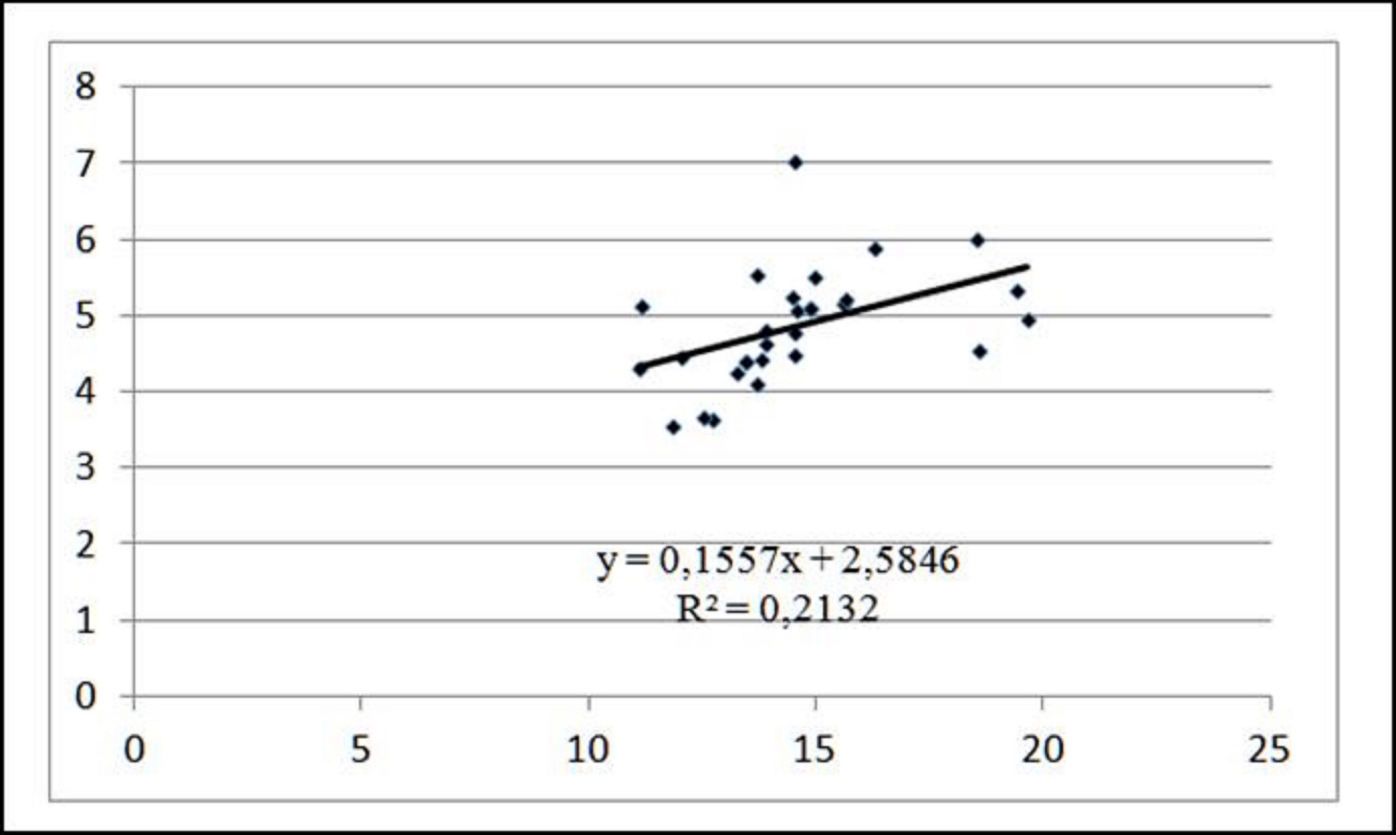
Linear approximation of the dependence of meat color on moisture holding capacity at ante-mortem live weight of 130 kg.

In pigs from the control and experimental groups, the link between color intensity and water holding capacity of meat was direct (*r*_*x,y*_ > 0) and statistically significant (*F*-value > *F*-critical) (Table 2). However, when animals were slaughtered at a weight of 110 kg, the association was apparent (0.5 < *r*_*xy110*_ < 0.7), and when animals were slaughtered at a weight of 130 kg, the relationship was modest (0.3 < *r*_*xy130*_ < 0.5). Thus, a rise in the meat’s redness indicates an increase in its water holding capacity across the entire research group. Additionally, we find that 21.32% of the variability in the redness of the meat in group II pigs was due to differences in their water holding capacity. The direct linear regression equation coefficient indicates that increasing the water- holding capacity of muscle fibers by 1.0% increased the degree of redness by 0.155 points on the Minolta L* scale in pigs weighing 130 kg (Fig. 4).

**Figure 5.**
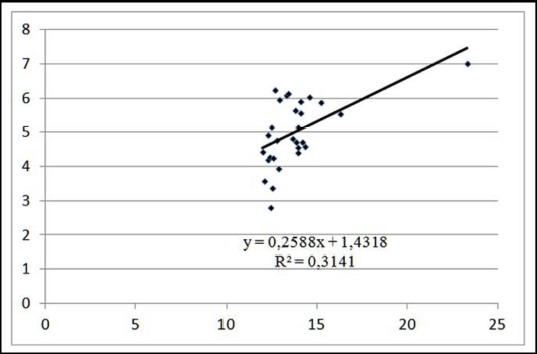
Linear approximation of the dependence of meat color on moisture holding capacity at ante-mortem live weight of 110 kg.

The coefficient of determination suggests that in meat from pigs weighing 110 kg before slaughter, the dynamics of red color darkening were created by changes in its water holding capacity by 31.41%, and by changes in its composition 68.59% (Fig. 5). The direct linear regression equation coefficient demonstrates that increasing the water holding capacity of meat by 1.0% results in an increase in its darkening by 0.258 points on the Minolta L* scale (Fig. 5) for 110 kg pigs.

The link between tenderness and marbling of the longest back muscle for pigs in both groups has distinct interpretations. Thus, the association between the indicators was moderate (0.3 < *r*_*xy130*_ < 0.5), inverse (*r*_*x,y130*_ < 0), and statistically significant (*F*-value 130 > *F*-critical 130) in pigs with a live weight of 130 kg. There was no statistically significant link between meat softness and marbling in peers with a pre-slaughter weight of 110 kg (*F*-value 110 *F*-critical 110) (Table 3).

The correlation showed no significant relationship between marbling and fat content for pigs of both groups.

Additionally, there was no statistically significant link between marbling and protein content in the examined pig population of both weight categories. The association between marbling and moisture content was direct and stable in both groups of pigs, but was greater in pigs 110 kg and evident in pigs 130 kg (Table 3).

**Table 2.** Indicators of correlation of a linear mathematical model by the method of least squares for the relationship between сolor intensity and pH and water holding capacity.

Indicators	Group I (110 kg)	Significance	Group II (130 kg)	Significance
pH_24_ in LL	−0.4604	***	−0.3278	***
Water holding capacity	0.5604	***	0.4617	***

LL – musculus longissimus lumborum.

*p < 0.05, **p <0.01, ***p <0.001.

**Table 3.** Indicators of correlation of a linear mathematical model by the method of least squares for the relationship between marbling and tenderness, fat content, protein content, and water content.

Indicators	Group I (110 kg)	Significance	Group II (130 kg)	Significance
Tendernes	−0.0694	NS	−0.3600	***
Fat content	−0.4570	NS	−0.2189	NS
Protein content	0.2569	NS	0.0697	NS
Water content	0.7234	***	0.6116	***

NS – not significant, LL – musculus longissimus lumborum.

*p < 0.05, **p <0.01, ***p <0.001.

**Figure 6.**
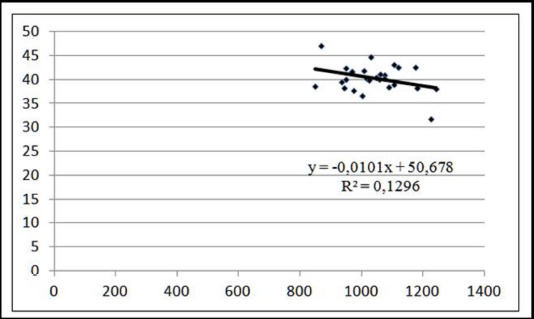
Linear approximation of the relationship between tenderness and marbling of meat at a pre-slaughter weight of 130 kg.

**Figure 7.**
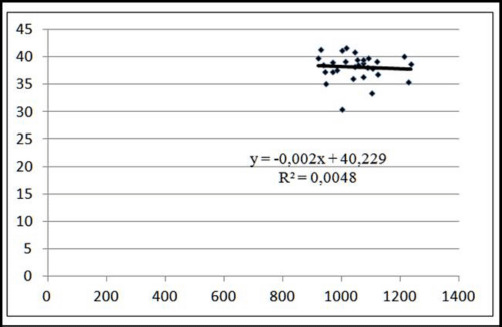
Linear approximation of the relationship between tenderness and marbling of meat at a pre-slaughter weight of 110 kg.

Variability in meat tenderness (12.96%) was driven by differences in the meat marbling of 130 kg pigs. When 1.0 points increased the marbling level on the Minolta L* scale, soreness decreased by 0.01 points (Fig. 6). The level of marbling had no significant effect on the softness of pigs weighing 110 kg before slaughter, indicating that an increase in marbling did not result in harsher meat (Fig. 7).

**Figure 8.**
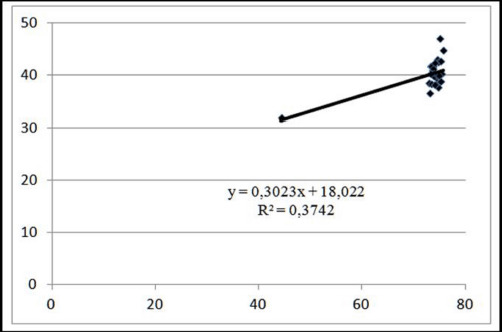
Linear approximation of the relationship between meat marbling and moisture content at pre-slaughter weight of 130 kg.

The coefficient of determination R2 indicated that a change in moisture content was associated with a 37.42% change in marbling in pigs weighing 130 kg. The marbling index improved by 1.0 points, which resulted in a 0.30% rise in the moisture content of the meat (Fig. 8).

**Figure 9.**
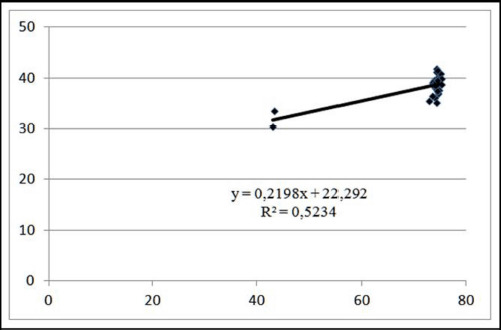
Linear approximation of the relationship between meat marbling and moisture content at pre-slaughter weight of 110 kg.

Simultaneously, the coefficient of determination between marbling and moisture content in the meat of 110 kg pigs indicates an interdependence of the parameters within 52.34%. Additionally, increasing the marbling value by 1.0 points on the Minolta L* scale increased the moisture content by 0.21%. The results are shown in Figure 9.

## Discussion

Correa et al. [28] did not confirm the absence of a pH variation between the meats of different weight categories of pigs. Additionally, the authors state that soluble collagen content decreased with increasing weight (*p* < 0.05), but we found that soluble collagen content increased with increasing carcass weight, which agrees with Skrlep et al. [29], who report a higher collagen content in heavy pig carcasses compared to light pig carcasses and a more developed connective tissue in the experiment’s pigs.

Our findings corroborate those of Sieczkowska et al. [30], who discovered that increasing the hot carcass weight above 90 kg has a beneficial effect on the rate and pH degree of longissimus dorsi muscles 45 min and 24 h after slaughter, resulting in increased water retention, decreased moisture loss, and less meat loss during cooking.

According to Choi et al. [31], greater live weights did not affect this investigation’s technological and sensory quality. He reported that no significant differences in muscle pH 4.5, lightness, drip loss, or shear force were observed across the groups (*p* > 0.05), and that increased live weight had no effect on sensory quality qualities such as tenderness, juiciness, or flavor. However, our results indicate that pH increases with carcass weight, as Latorre [32] found that the pH of pigs slaughtered at 133 kg was significantly higher (*p* < 0,05) than the pH of pigs slaughtered at 116 or 124 kg. Additionally, our findings contradict Auqui’s [17] report, who identified no weight-dependent impacts on carcass quality (carcass yield, length, or back fat thickness) or on moisture, pH, or the L* and a* coordinates.

At the same time, our findings do not corroborate with the report of Tibau et al. [33] and Zullo et al. [34], who observe that protein and fat content increase with increasing pre-slaughter weight. Tibau et al. [33] showed that the pork loin’s tenderness and redness rose as the pre-slaughter weight increased. Our investigation did not confirm this conclusion. Increased pre-slaughter weight resulted in a drop in ash levels, an increase in protein content, an increase in fat content, and a decrease in moisture content [34]. However, we found no correlation between our findings and their premise. According to Wu et al. [1], moisture loss was less in hefty pig carcasses (182 kg). However, we were unable to locate confirmation of its findings. Also, the statements of Moon et al. [35] and Latorre [32] about increase in the intensity of the red color of the meat with increasing pre-slaughter weight did not coincide with the data we obtained. Our pH estimations are inconsistent with those reported by Moon et al. [35], who argues that increasing the pre-slaughter weight of pigs from 95 to 125 kg may improve pork quality. The authors discovered that pH (pH limit) decreased when pre-slaughter weight increased, but no variation in carcasses weighing 125 kg or more was detected. Our findings corroborate the report of Durkin et al. [36], who claims that pigs slaughtered at a heavier weight (140 kg) have a higher pH in muscle than pigs slaughtered at a lighter weight (120 kg).

Skrlep et al. [29] concluded that an increase in fat content and a decrease in the water-to-protein ratio as a result of increasing age and pre-slaughter weight should benefit meat quality. In our investigations, these markers did not depend on the pre-slaughter weight, which contradicts this researcher’s assertion.

Our findings contradict Bertol et al.’s [37] report, claiming that meat from heavier pigs had a more vivid red color and the same intramuscular fat level as meat from lighter pigs, which contradicts our findings. In both his and our tests, increasing pre-slaughter weight resulted in a modest reduction in meat softness. Jeong et al. [38] reported the same thing, stating that while the redness of the loin rose with increasing pre-slaughter weight (*p* < 0.05), other physico-chemical features were unaffected. On the contrary, our findings indicate that the severity of the meat’s redness is not dependent on its pre-slaughter weight.

Our findings corroborate those of Hwang et al. [39], who discovered that when pigs’ pre-slaughter weight increased, meat color intensity and taste sensitivity increased dramatically (*p* < 0.05), while softness reduced significantly (*p* < 0.001). Pre-slaughter weight correlated positively with flavor but negatively with softness, as we also discovered in our trial. Our findings contradict those of Lukac et al. [40], who highlights that the maximum protein and water content was found in the ham and shoulder of animals weighing 100–110 and 111–120 kg pre-slaughter, respectively, and the lowest quantity was found in the neck of animals weighing 121–130 kg pre-slaughter. The maximum intramuscular fat level was observed in animals weighing 120–130 kg, while the lowest was observed in animals weighing 100–110 kg. A substantial difference between animals with various pre-slaughter weights was not demonstrated using the pH value, corroborating our findings. Ba et al. [41] advanced a similar thesis, stating that a group of pigs weighing 120 kg had a larger fat content and the ability to retain water than a group of pigs weighing 100 or 110 kg (*p* < 0.05). We obtained a variety of responses from these indicators.

Our findings contradict Imrich et al.’s [42] assertion that a higher pre-slaughter weight has a beneficial influence on the color of the meat, as pigs weighing more than 110 kg obtain a much lower L* value than pigs of lower weight. He viewed the rise in protein content in meat as a beneficial impact of increased weight prior to slaughter, as pigs weighing more than 100 kg have a significantly greater protein content than pigs weighing less than 100 kg. We were unable to locate any evidence to corroborate his allegation.

Additionally, our findings contradict those of Harsh et al. [43], who discovered that the color intensity of the longest back muscle increased (*p* < 0.001) as pig pre-slaughter weight increased, but the pH declined (*p* < 0.001). However, changes in pre-slaughter weight of only 1.23% resulted in statistically significant differences in pH. However, according to the results of our investigation, the pH value increased as pre-slaughter weight increased.

Additionally, our findings contradict those of Kondratov [44], who reports varied pH values in the meat of pigs with varying pre-slaughter weights 48 h after slaughter, namely 5.81–5.90 for a 100 kg of pig, 5.70–5.92 for a 120 kg pig, and 5.70–5.86 for a 140 kg pig.

Our findings contradicted Litwin Czuk et al.’s [45] observation that, in general, increased marbling of beef is associated with a decrease in water and protein content, which contradicted our findings. He corroborated this discovery by observing a positive link between meat marbling and fat content and a negative correlation between meat marbling and water and protein content.

According to Piao et al. [46], the water retention capacity of 110 kg market weight pigs was the greatest. Pigs weighing 100 kg had lower juiciness, softness, shear forces, and total palatability than pigs weighing more than 100 kg (*p* < 0.01). However, we found no effect of pre-slaughter weight on water holding capacity in our study, which contradicts the report of Elbert [11], whose research indicated that the heavy weight (120 kg) group had a higher fat content and water holding capacity than the light weight (100 kg) and medium weight (110 kg) groups (*p* < 0.05).

## Conclusion

It was discovered that meat from the animals slaughtered at 130 kg had an increase in the following indicators: marbling – by 2.0 points on the Minolta L* scale or 5.24% (*p* < 0,01), active acidity pH in the muscles of the ham – by 0.20 pH or 3.57% (*p* < 0.01) and in the longest muscle of the back – by 0.10 pH or 1.82% (*p* < 0.001), and the content of soluble collagen – by 0%.

There were no statistically significant differences in the water holding capacity, color intensity, active acidity, EC, free and bound moisture content, fat content, and protein content from pigs slaughtered at 110 and 130 kg.

The pre-slaughter weight factor had a significant effect on the index of active acidity pH in ham muscles 24 h after slaughter, at 10.35%, and on the marbling of the meat on the longest back muscle cut of pigs, at 13.31%.

A stronger inverse linear relationship between the intensity of meat color and the level of active acidity pH and its water holding capacity in the longest muscle of the back in animals with a pre-slaughter weight of 110 kg compared to peers slaughtered at 130 kg, which resulted in a faster darkening of meat and an increase in its water holding capacity with the same decrease in pH in these animals, in our opinion.

A moderate, negative association was discovered between the softness and marbling of the longest back muscle in pigs slaughtered at 130 kg, demonstrating that as pigs gain weight up to 130 kg, meat marbling increases, and tenderness decreases. The reliability of the link between these variables was not proven at 110 kg slaughter.

Further research into the physico-chemical properties of the longest back muscle in pig carcasses is advised, emphasizing the effect of postnatal nutrition and husbandry practices on pigs.

## List of Abbreviations

mg = Milligram, gm = Gram, kg = Kilogram, m^2^ = Meter square, cm^2^ = Centimeter square, % = Percentage, °C = Degrees Celsius, LLC = Limited liability company, NS = Not significant, LL = musculus longissimus lumborum, EC = electrical conductivity, WHC = water holding capacity, UF = universal forced, AS = automatic scales, DKL = digester Kjeldahl long, UDK = universal digester Kjeldahl, SER = solvent extractor Randall.
